# The Bidirectional Relationship between Quality of Life and Eating Disorder Symptoms: A 9-Year Community-Based Study of Australian Women

**DOI:** 10.1371/journal.pone.0120591

**Published:** 2015-03-26

**Authors:** Deborah Mitchison, Alexandre Morin, Jonathan Mond, Shameran Slewa-Younan, Phillipa Hay

**Affiliations:** 1 School of Medicine, University of Western Sydney, Sydney, Australia; 2 Institute for Positive Psychology and Education, Australian Catholic University, Sydney, Australia; 3 Department of Psychology, Faculty of Human Sciences, Macquarie University, Canberra, Australia; 4 Research School of Psychology, Australian National University, Sydney, Australia; 5 Centre for Health Research, School of Medicine, University of Western Sydney, Sydney, Australia; 6 School of Medicine, James Cook University, Townsville, Australia; University Hospital of Bellvitge-IDIBELL; CIBER Fisiopatología Obesidad y Nutrición (CIBERObn), Instituto Salud Carlos III; Department of Clinical Sciences, School of Medicine, University of Barcelona, Spain, SPAIN

## Abstract

**Objective:**

Studies that have investigated quality of life (QoL) in eating disorders (EDs) have been focussed on the impact of the ED on QoL and little is known regarding the possible reciprocal impact of QoL on EDs. The aim of this study was to provide a first-time investigation of possible bidirectional relationships between EDs and both health-related QoL (HRQoL) and psychological distress (PD).

**Method:**

Structural equation modeling was applied to longitudinal data collected from a community sample of Australian women (*N* = 828) surveyed at baseline, five annual follow-ups, and again after nine years. Participants reported height and weight (from which body mass index, BMI, was calculated) and completed measures of ED symptoms (Eating Disorder Examination Questionnaire), HRQoL (12-item Medical Outcomes Study Short Form), and PD (Kessler Psychological Distress Scale).

**Results:**

Overall, evidence was found for a bidirectional relationship, whereby ED symptoms predicted reduced HRQoL and greater PD over time, while lower levels of HRQoL and greater PD in turn predicted increased levels of ED symptoms. These relationships were stable, observable within 12 months, and remained observable over a time period of at least four years. However, also observed were some inconsistent findings where ED symptoms predicted a short term (one year) improvement in mental HRQoL. This short term boost was not sustained at longer follow-ups.

**Conclusions:**

Not only do ED symptoms impact on HRQoL and PD, but perceived poor HRQoL and PD also contribute to ED symptom development or exacerbation. This supports a movement away from symptom-centric approaches whereby HRQoL is conceptualized as a passive outcome expected to be rectified by addressing ED symptoms. Improvement in QoL and PD might rather be viewed as targets to be pursued in their own right under broader approaches in the treatment of EDs.

## Introduction

The diagnostic criteria for eating disorders (EDs) in the *Diagnostic and Statistical Manual of Mental Disorders* (DSM-5 [1]) differ from that of other mental disorders in that symptom-related distress and functional impairment is not included as a distinct and necessary criterion for diagnosis. This is in contrast to almost all other disorders in the manual where it is essential that the symptoms “*cause clinically significant distress or impairment in social*, *occupational*, *or other important areas of functioning*” (e.g., Obsessive Compulsive Disorder, p.237 [1]). Recognition of functional impairment and/or distress within the EDs may be on the rise however, as the past decade has witnessed an increase in studies investigating closely related constructs and their relationship to features of EDs (for a recent review see [2]).

Quality of life (QoL) is one such construct, and is defined by the World Health Organisation as “*…a broad ranging concept affected in a complex way by the person's physical health*, *psychological state*, *level of independence*, *social relationships*, *personal beliefs and their relationship to salient features of their environment*.*”* (p153 [3]). The concept of QoL thus generally takes into consideration a number of key life domains, such as mental and physical health, relationships, occupation, education, leisure, spirituality, and community engagement. Health-related QoL (HRQoL) measures, such as the Medical Outcome Studies Short Forms [4,5] assess the extent to which an individual perceives that their health impacts on their level of functioning within domains such as those listed above. However, although they measure aspects of mental HRQoL, these measures do not account for psychological distress in general, which as noted above is another critical component of major mental disorders [1] and QoL definitions [3]. Further, the distinction made by the DSM between psychological distress and functional impairment in defining *disorder* is similarly paralleled by distinctions made in the definition of *wellbeing* (i.e. the absence of disorder), which has both hedonic (related to a lack of distress and the presence of positive states) and eudaimonic (related to optimal functioning) components [6]. Thus alongside HRQoL in this study, we also consider psychological distress as a separate and key component of QoL.

Reviews of the literature on QoL and EDs conclude that reduced QoL is associated not only with ED diagnoses but also with subclinical levels of disordered eating and specific ED symptoms and features [2,7,8]. Thus far, this literature has almost exclusively focused on the impact of disordered eating on QoL, and little is known about the inverse of this relationship—the impact of QoL on the development, exacerbation, or maintenance of ED pathology. This is highlighted in the development of the first five ED-specific measures of HRQoL [9,10,11,12,13], all of which assume a uni-directional relationship, whereby participants rate how their ED has affected QoL in various life domains.

Although the current literature (summarised in the reviews above) on the ED-QoL relationship is consistent with the position that EDs result in reduced QoL, an equally consistent explanation of these cross-sectional findings is that poor QoL promotes disturbances in eating and body image. This latter explanation is plausible if egosyntonic ED symptoms (e.g., unhealthy weight loss strategies such as fasting and excessive exercise) are conceptualised as being adopted in order to enhance happiness and self-esteem not provided through other life domains [14]. Conversely, egodystonic symptoms (e.g., binge eating, purging) may be used to self-soothe or cope with perceived ineffectiveness in life domains such as interpersonal functioning. Thus poor QoL may predispose one to develop or exacerbate ED symptoms. On the other hand, perhaps the most plausible (and thus far untested) explanation is that the relationship between QoL and ED pathology is bidirectional; that is, ED symptoms result in reduced QoL, and at the same time poor perceived QoL promotes the development or exacerbation of ED symptoms.

In order to test such directional hypotheses, analysis of longitudinal data is necessary. Other than an indication of the directionality of the associations between constructs, data collected over multiple time points may also provide additional and highly valuable information regarding [15]: (i) the minimum time needed for a relationship to be detected, (ii) the stability of a relationship (whether a specific relationship is replicable over different time periods), and (iii) the longevity of any observed relationships (the maximum time over which a relationship can be detected). In the only longitudinal community study of the relationship between EDs and HRQoL found to date, Wade and colleagues found that subclinical ED symptomatology at baseline was associated with poorer HRQoL three, six, and nine years later in a large sample of Australian women [16]. This provided evidence that the negative impact of ED pathology on HRQoL can be observed within three years, is stable across time, and lasts for at least nine years. However data on ED pathology was not available past baseline, and as such the impact of HRQoL on ED pathology could not be explored.

Although translational research has seen QoL become regularly considered in the treatment evaluation process [2], it still remains largely outside of therapeutic intervention itself. A recent exception is provided in a randomized controlled trial comparing versions, modified for severe and enduring (SE) illness, of cognitive behavioural therapy (CBT-SE) and specialist supportive clinical management (SSCM-SE) in the treatment of chronic anorexia nervosa [17]. The therapies were adjusted such that improvement in QoL, rather than the reduction of ED symptoms, was posited as the main aim of treatment. Results indicated that both CBT-SE and SSCM-SE were associated with improved HRQoL by the end of treatment, as well as a reduction of ED symptoms, including modest weight gain, despite this not being a primary outcome. This study, which was conducted with patients with chronic anorexia nervosa—perhaps the most difficult to treat of all ED subgroups—suggests that placing a greater emphasis on QoL improvement could possibly represent a pathway to reducing ED symptoms among the broader population of people presenting with eating and body image disturbances. A critical step in the verification of this possibility involves a longitudinal analysis of the relationships between QoL and ED pathology, taking into account their possible bidirectional relations, preferably in a community-based sample [18,19].

### Aims

The objective of this study was to investigate the putative bidirectional relationships between ED pathology and QoL (i.e. HRQoL and psychological distress) using longitudinal data obtained from a community sample of women. A further objective was to assess the stability and longevity of the observed relationships, as well as the minimal time interval required before the relations could be observed. This was to be achieved by examining whether ED pathology and QoL were significant predictors of each other over time lags of one, two, and four years, using autoregressive cross lagged analyses conducted within a structural equation modelling framework. It was hypothesized that we would find evidence for a bidirectional model where we would not only confirm that ED pathology contributes to reduced QoL; but also find new evidence that poor QoL predicts increased ED pathology, and that these relationships are persistent over time.

## Method

### Participants

To purposively over-sample for ED symptoms, our full sample includes participants recruited from a university/college community subsample as well as from an ED-symptomatic community subsample. Both subsamples were recruited at the same time, and are composed of mainly young women who completed a health survey with a specific focus on EDs.

The university/college subsample was recruited through advertisements in four regional universities and colleges in the Australian states of Queensland and Victoria (see [20]). Seven hundred and six participants returned the surveys and agreed to take part in the longitudinal study. Due to the method of recruitment, the response rate was impossible to estimate. There was no age cut-off for participant selection, however 86.6% of participants were younger than 42 years of age, with a further 9.7% being 43 to 50 years and 4.7% being 51 to 67 years of age. The ED symptomatic subsample was identified from a larger group of 5255 (response rate 52.6%) participants randomly selected from the Australian Capital Territory (ACT) electoral roll between the ages of 18 and 42, who responded to a postal survey. An analysis of pilot data found no evidence of non-response bias [21]. The sample represented approximately 10% of all female residents aged 18 to 42 in the greater ACT region, and were representative of this population in all demographic features assessed [22]. In a follow-up interview of the participants who had scored > 2.5 on the global scale of the *Eating Disorder Examination Questionnaire* (EDE-Q; see below) and endorsed at least one ED behaviour (objective or subjective binge eating, purging, compulsive exercise, and/or severe dietary restriction), 122 were identified as having ED symptoms of ‘clinical severity’ and agreed to join the longitudinal study [23]. Clinical severity was defined as endorsing extreme weight/shape concerns and/or engaging in one or more ED behaviours (binge eating, purging, compulsive exercise, and/or severe dietary restriction) on at least a weekly basis over the previous three months. For additional details on the recruitment and selection of participants see Mitchison, Crino, and Hay [24].

At baseline, participants’ mean age was 27.7 years (SD = 10.2; range = 18 to 67) and mean BMI was at the upper end of the healthy range [25], 24.8 kg/m^2^ (SD = 5.6; range = 17.3 to 54.8). The majority of participants were born in Australia (98.9%) and were not married or in a defacto relationship (71.2%). Participants at the year-9 follow-up were on average older than participants who dropped out (M = 37.8 years at baseline; SD = 10.6; range = 27 to 74) and had a higher average BMI (M = 26.8 kg/m^2^; SD = 6.4; range = 16.9 to 53.6). Baseline demographic comparison of the two subsamples demonstrated no significant difference in age (*p* = 0.34) or migrant status (*p* = 0.23) across subsamples (university/college: *M* = 27.52, *SD* = 10.77; 98.7% Australian born; ACT electoral roll: *M* = 28.48 years, *SD* = 6.34; 100% Australian born). However the university/college subsample (27.2%) were less likely than the ACT electoral roll subsample (37.7%) to be married [*χ* ^2^ (1) = 5.60. *p* = 0.02].

The two subsamples (combined baseline *N* = 828) were assessed in tandem by members of the research team annually for a period of five years, and then once more nine years following baseline assessment. All participants who returned surveys at baseline were either posted or emailed (according to their preference) follow-up surveys up to nine years later. Non-responding participants at each follow-up were re-sent the survey up to three additional times over a six month period to maximise the response rate. Of the 828 baseline participants, 54.1% (*n* = 448) completed the first follow-up, 54.1% (*n* = 448) completed the second follow-up, 53.5% (*n* = 443) completed the third follow-up, 52.4% (*n* = 434) completed the fourth follow-up, and 44.0% (*n* = 364) completed the year 9 follow-up. At least two surveys were returned by 70.8% of participants, at least three by 61.6% of participants, at least four by 54.2% of participants, at least five by 42.4% of participants, and all six by 25.8% of participants.

The study was reviewed and approved by the human research ethics committee at the University of Western Sydney (ethics approval number: H9283). Written informed consent was obtained at baseline and each of the follow-up time-points by requesting potential participants to read an information statement and if agreeing to take part in the study, to return a signed consent form with the completed survey.

### Measures

The baseline and follow-up surveys included questions on demographic information, psychopathology, QoL, and health service utilisation. For the present study, completed responses to the following specific measures were used. Estimates of scale score reliability proved fully satisfactory (0.835 to 0.995) and are provided in S1 Analyses. Correlations between all constructs are also reported in this file.

#### Eating Disorder Symptoms

The 22 items of the Eating Disorder Examination Questionnaire (EDE-Q) [26,27] that load onto its four subscales (Dietary Restraint, Eating Concerns, Weight Concerns, Shape Concerns) were used as indicators of the latent construct of ED pathology (abbreviated to ED in the analyses below). The questionnaire assesses ED pathology over the past month and scores range from 0 to 6 with higher scores indicating greater ED severity. The EDE-Q has been validated in community and clinical ED samples. Norms for Australian women have been reported previously, together with evidence of adequate scale score reliability (α coefficients ≥ 0.8) and case predictive validity (sensitivity = 0.8, specificity = 0.8) [28]. As expected, given that the ACT electoral roll subsample was selected based on the presence of ED symptoms, their baseline scores on the EDE-Q were on average higher than the levels observed in the university/college subsample (see Table 1).

**Table 1:** Eating Disorder Pathology and Health-Related Quality of Life at Baseline.

	University/College (*n* = 706)	ACT Electoral Roll (*n* = 122)
Eating Disorder Examination Questionnaire (EDE-Q)		
Restraint Subscale	1.63 (1.51)*	3.24 (1.42)
Eating Concerns Subscale	1.03 (1.28)*	2.95 (1.21)
Weight Concerns Subscale	2.16 (1.60)*	4.13 (0.91)
Shape Concerns Subscale	2.51 (1.63)*	4.79 (0.86)
Body Mass Index (kg/m^2^)	24.37 (5.23)*	27.02 (6.85)
Medical Outcomes Short Form (SF-12)		
Mental Health Summary Scale	44.41 (11.36)	42.44 (8.17)
Physical Health Summary Scale	52.17 (7.57)*	43.12 (6.34)
Kessler Psychological Distress Scale (K-10)	18.75 (7.23)*	38.13 (7.41)

*Statistically significant difference between the ACT Electoral Roll and University/College samples, *p* < 0.001.

In order to obtain a more complete picture of ED symptoms, body mass index (BMI) is also included in this study, and treated as an observed construct in the analyses. BMI is an aspect of ED pathology [1] not measured in the EDE-Q subscales and is impacted by ED behaviors such as dietary restriction and binge eating. Thus BMI was considered an important additional indicator of ED symptoms. To assess BMI, participants were asked to report their current height and weight and this was converted into BMI using the algorithm, kg/m^2^. Higher BMI indicates higher body weight relative to height. In comparison to objective anthropometric measurements, self-reported height and weight tend to be slightly less reliable [29], but nonetheless remain the most common method used in large postal community surveys, such as in the present study. Further, the tendency to slightly underestimate weight and overestimate height is consistent both within and across studies, which supports its usefulness and comparability [29].

#### Health-Related Quality of Life

The 12 items of the SF-12 [30] were used to assess physical and mental HRQoL (referred to as PHQoL and MHQoL, respectively, in the analyses below). The SF-12 is a standardized measure of HRQoL and has been used extensively in studies of both physiological and psychological health conditions. More precisely, the items assess limitations in physical activities, pain, anxiety, depression, vitality, and the impact of physical and emotional health on social functioning and productivity in work and other important roles. Higher scores indicate higher levels of functioning. Adequate psychometric properties have been demonstrated in many populations, including an Australian population sample [30,31].

#### Psychological Distress

The 10 items from the Kessler Psychological Distress Scale (K-10) [32] were used to assess psychological distress (referred to as PD in analyses below). The K-10 was developed specifically for general population samples. Higher scores indicate greater distress. The K-10 has been found to have excellent scale score reliability (α coefficient = 0.93) in a general population sample of US adults [32], and to be more predictive of anxiety and mood disorders than other screening instruments (e.g., the General Health Questionnaire [33]) in a general population sample of Australian adults [34].

### Data Analysis

#### Estimation

To take into account the ordered-categorical nature of the answer scales used to assess the constructs considered in this study (7 categories reflecting frequency with a dominance of 0 for the EDE-Q; 2 to 6 categories for the SF-12; 5 categories for the K-10), all models were estimated using Mplus 7.0’s robust weight least square estimator (WLSMV) [35]. WLSMV has been found to outperform Maximum Likelihood (ML) with ordinal ratings scales similar to those used in this study [36,37,38,39]. To account for missing responses or time points, models were estimated based on the full available information, based on algorithms implemented in Mplus for WLSMV [40]. This procedure has comparable efficacy to multiple imputation, while being more efficient [41,42,43], under Missing At Random (MAR) assumptions, and even sometimes to violations of this assumption. With WLSMV, missing data are allowed to be conditional on all observed and latent variables included in the model, which includes the constructs themselves at preceding time points in this study.

#### Model Fit

Model fit was evaluated using [44,45]: the WLSMV Chi-square statistic (χ²), the Comparative Fit Index (CFI), the Tucker-Lewis Index (TLI), the Root Mean Square Error of Approximation (RMSEA) and its 90% confidence intervals. Values greater than. 90 and. 95 for the CFI and TLI respectively reflect adequate and excellent fit, while values smaller than. 08 or. 06 for the RMSEA respectively indicate acceptable and excellent fit. With WLSMV, Chi-square difference tests are conducted via Mplus’ DIFFTEST function, MDΔχ^2^ [46,47]. However, as with the χ^2^, MDΔχ^2^ also tends to be oversensitive to sample size and to minor misspecifications. Thus, additional indices are generally used when comparing nested models, such as in a sequence of measurement invariance tests [48,49]. A more constrained model can be considered as providing an equivalent fit than a less constrained model when it is accompanied by CFI decline of. 01 or less and by a RMSEA increase of. 015 or less [48,49].

#### Predictive Models

**Fig 1:**
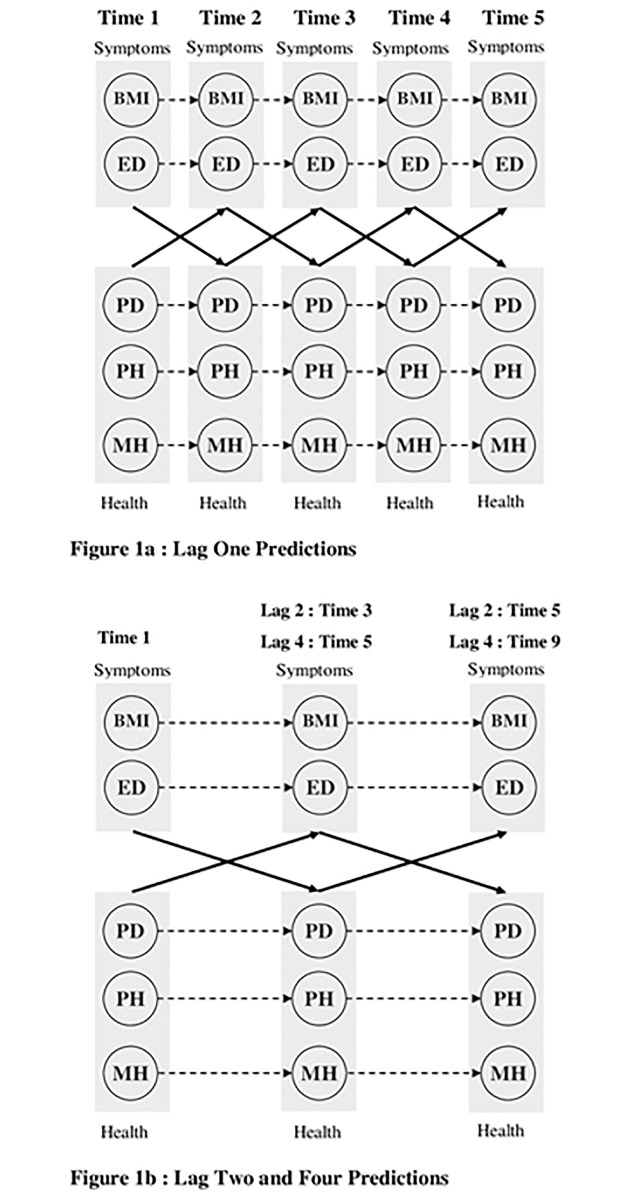
Predictive relations between eating disorders symptoms and health indicators. Note. BMI = Body Mass Index; ED = Eating Disorder symptoms; PD = Psychological Distress; PH = Physical Health-Related Quality of Life; MH = Mental Health-Related Quality of Life. Constructs are represented by circles. All constructs, except BMI, are fully latent and estimated directly from the items answered by the participants (and thus are controlled for measurement error). The dashed arrows reflect the autoregressive paths whereby each construct at Time t predicts itself at Time t+1. Sets of constructs relating to either symptoms of eating disorders (ED and BMI) or health indicators (PD, MH, and PH) are grouped together in greyscale boxes so that all constructs within a box are specified as predicting all constructs within the other box. The full arrows reflect these cross lagged relations reflecting the reciprocal effects of eating disorders symptoms on health indicators, and of health indicators on eating disorders symptoms. All constructs are specified as correlated with one another within time.

Using confirmatory factor analyses (CFA), we first verified the adequacy of the a priori longitudinal measurement model across all time waves, as well as its measurement invariance across time wave. The results from these CFA, which are fully reported in S1 Analyses, supported the adequacy of the measurement model and its measurement invariance across time waves. The measurement part of the predictive models was specified as invariant based on the results from these preliminary CFA to ensure stable measurement over time, as well as to achieve greater stability in the models due to greater parsimony. The predictive models tested here are illustrated in Fig. 1, where circles represent the constructs. The measurement part of the model at the item level are not included in the figure for greater clarity. The measures of ED and BMI (i.e., the symptom-related constructs) are treated together to avoid cluttering the figure as the paths linking them to the other constructs are fully parallel. For the same reason, the three measures of PD, MHQoL, and PHQoL (i.e., QoL-related constructs) are also treated together. Each predictive model was first estimated without any constraints imposed on the predictive paths. These models are autoregressive cross lagged models [50,51]. In the autoregressive part, each construct measured at Time t predicted itself at Time t+1. This is illustrated in Fig. 1 by the dotted arrows. In the cross lagged part, each group of constructs measured at Time t were allowed to predict the other group of constructs at Time t+1 (the bold arrows). Thus, QoL-related constructs were allowed to predict symptom-related constructs measured at the next time point, while the symptom-related constructs were also allowed to predict QoL-related constructs measured at the next time point. All other longitudinal regressions/correlations were constrained to be zero, but correlations between constructs were freely estimated at each time point.

#### Predictive equilibrium

A critical assumption of longitudinal models is that the predictive system has reached equilibrium (e.g., [15]). This assumption tests whether the overall pattern of associations between constructs is stable across time periods—supporting the idea that the results are not a function of time-specific events and can be expected to generalize to different time periods. To test his assumption, we re-estimated these models while adding equality constraints on the predictive paths across time periods. Thus, the relations estimated between a specific pair of constructs across the first time interval (Time t to Time t+1) was constrained to be equal to the same relation estimated between these same constructs across the next intervals (Time t+1 to t+2, t+2 to t+3, etc.). An objective of this study was to test if the relations changed as a function of the time lag in order to systematically investigate the latency of the relations. Thus, these analyses were first conducted for a time lag of 1 year using waves 1 to 5 of the study (Fig. 1a). Then similar analyses were conducted for a time lag of 2 years using waves 1, 3 and 5 of the study, and for a time lag of 4 years, using waves 1, 5, and 9 (Fig. 1b). To take into account the number of different models estimated, finding were considered significant at *p* < 0.01.

## Results

The fit results from the predictive models are reported in Table 2. These results show that these models also provide a fully acceptable level of fit to the data (RMSEA ≤. 06; CFI ≥. 90; TLI ≥. 90). The results also show that the predictive system has reached equilibrium across time points, as illustrated by changes in fit indices that remain well below the recommended cut-off scores of. 01 for the CFI and. 015 for the RMSEA.

**Table 2:** Results from the predictive models tested in this study.

*Models*	*χ²*	*df*	*RMSEA (CI)*	*CFI*	*TLI*	*MDΔχ²*	*Δdf*	*ΔRMSEA*	*ΔCFI*	*ΔTLI*
*Predictive Models*, *One Year Lag (Times 1 to 5)*										
Predictive Model (lag 1)	37110.893*	24952	.024 (.024-.025)	.919	.922	—	—	—	—	—
Invariant Predictive Model (lag 1)	37647.763*	25003	.025 (.024-.025)	.916	.919	461.813*	51	+.001	-.003	-.003
*Predictive Models*, *Two Year Lag (Times 1–3–5)*										
Predictive Model (lag 2)	15061.290*	8774	.029 (.029-.030)	.938	.939	—	—	—	—	—
Invariant cross lagged model (lag 2)	15243.080*	8791	.030 (.029-.031)	.937	.938	149.509*	17	+.001	-.001	-.001
*Predictive Models*, *Four Year Lag (Times 1–5–9)*										
Predictive Model (lag 4)	14070.069*	8774	.027 (.026-.028)	.946	.947	—	—	—	—	—
Invariant cross lagged model (lag 4)	13973.726*	8791	.027 (.026-.028)	.947	.948	58.165*	17	.000	+.001	+.001

*Note*. *χ²* = WLSMV chi square; *df* = degrees of freedom; *RMSEA* = Root mean square error of approximation; *CI* = 90% Confidence Interval for the RMSEA; *CFI* = Comparative fit index; TLI = Tucker-Lewis index; Δ since previous model; MDΔχ^2^: chi square difference test based on the Mplus DIFFTEST function for WLSMV estimation. With WLSMV estimation, the χ^2^ values are not exact, but "*estimated*" as the closest integer necessary to obtain a correct *p*-value. This explains why sometimes the χ^2^ and resulting CFI values can be non-monotonic with model complexity. Given that the MDΔχ^2^ tends to be oversensitive to sample size and to minor model misspecifications, as the chi-square itself, and to take into account the overall number of MDΔχ^2^ tests used in this study, the significance level for these tests was set at. 01 [52,53,54].

* *p* < 0.01.

### Results Based on Lag 1 analyses

The detailed parameter estimates from the predictive models estimated across a one-year time lag are reported in Table 3 (additional technical details on the estimation of the one-year lag models are provided in S1 Analyses). In regards to the relationships between ED pathology and HRQoL, as hypothesised, higher levels of ED symptoms and higher BMI independently predicted poorer PHQoL after one year. Unexpectedly however, ED symptoms and higher BMI independently predicted improved MHQoL one year later. Examining the inverse relationships, our hypotheses were supported, with poorer PHQoL predicting higher levels of ED symptoms and higher BMI one year later. Poorer MHQoL also predicted higher levels of ED symptoms one year later, but had no effect on BMI. In regards the relationships between PD and ED pathology, as hypothesised, higher levels of ED symptoms and BMI predicted greater PD one year later, while greater PD predicted higher levels of ED symptoms one year later but had no effect on BMI. For more information, the *R* ^2^ associated with the various predictive models considered here are reported in S1 Analyses. Overall, the *R* ^2^ (*M* = 54.1%; range: 25.4% to 89.8%) remain relatively high and show more similarities across times waves and time lags than differences, although they do show a slight tendency to decrease across time lags (Lag 1: *M* = 59.1%, range: 32.7% to 89.8%; Lag 2: *M* = 50.23%, range: 25.4% to 70.1%; Lag 4: *M* = 47.9%, range: 28.0% to 73.4%).

**Table 3:** Standardized parameter estimates from the final predictive models (One Year Lag).

*One Year Lag*		*Time 1 → Time 2*	*Time 2 → Time 3*	*Time 3 → Time 4*	*Time 4 → Time 5*	*Time t → Time t+1*
*Predictors (t)*	*Outcomes (t +1)*	*ß (S*.*E*.*)*	*ß (S*.*E*.*)*	*ß (S*.*E*.*)*	*ß (S*.*E*.*)*	*ß (S*.*E*.*)*
BMI	BMI	0.833 (0.013)**	0.930 (0.008)**	0.899 (0.009)**	0.900 (0.010)**	0.875 (0.010)**
	PD (K-10)	0.079 (0.031)	0.065 (0.026)	0.056 (0.022)	0.055 (0.022)	0.092 (0.036)
	PHQoL (SF-12)	-0.040 (0.010)*	-0.047 (0.011)*	-0.048 (0.011)*	-0.047 (0.011)*	-0.052 (0.012)*
	MHQoL (SF-12)	0.075 (0.021)*	0.083 (0.023)*	0.088 (0.025)*	0.084 (0.023)*	0.140 (0.039)*
ED (EDE-Q)	ED (EDE-Q)	0.308 (0.013)*	0.631 (0.016)*	0.698 (0.025)*	0.604 (0.026)*	0.503 (0.018)*
	PD (K-10)	0.451 (0.031)*	0.442 (0.032)*	0.511 (0.035)*	0.587 (0.037)*	0.584 (0.038)*
	PHQoL (SF-12)	-0.086 (0.007)*	-0.119 (0.010)*	-0.163 (0.014)*	-0.190 (0.015)*	-0.124 (0.010)*
	MHQoL (SF-12)	0.262 (0.019)*	0.342 (0.024)*	0.492 (0.033)*	0.547 (0.035)*	0.543 (0.036)*
PD (K-10)	PD (K-10)	0.653 (0.014)*	0.613 (0.020)*	0.534 (0.016)*	0.549 (0.015)*	0.510 (0.011)*
	BMI	-0.015 (0.009)	-0.018 (0.012)	-0.018 (0.012)	-0.019 (0.012)	-0.010 (0.007)
	ED (EDE-Q)	0.129 (0.012)*	0.252 (0.020)*	0.210 (0.019)*	0.163 (0.016)*	0.127 (0.011)*
PHQoL (SF-12)	PHQoL (SF-12)	0.628 (0.014)*	0.705 (0.014)*	0.713 (0.013)*	0.709 (0.014)*	0.692 (0.011)*
	BMI	-0.074 (0.011)*	-0.079 (0.012)*	-0.077 (0.012)*	-0.077 (0.012)*	-0.067 (0.010)*
	ED (EDE-Q)	-0.089 (0.011)*	-0.148 (0.018)*	-0.121 (0.015)*	-0.089 (0.012)*	-0.112 (0.014)*
MHQoL (SF-12)	MHQoL (SF-12)	0.521 (0.015)*	0.524 (0.017)*	0.586 (0.015)*	0.496 (0.015)*	0.542 (0.012)*
	BMI	-0.003 (0.013)	-0.003 (0.014)	-0.003 (0.014)	-0.003 (0.012)	-0.002 (0.008)
	ED (EDE-Q)	-0.155 (0.015)*	-0.244 (0.021)*	-0.210 (0.020)*	-0.138 (0.014)*	-0.127 (0.012)*

*Note*. BMI = Body Mass Index; ED (EDEQ) = Eating Disorder pathology (Eating Disorder Examination Questionnaire); PD (K-10) = Psychological Distress (Kessler Psychological Distress Scale); PHQoL (SF-12) = Physical Health-Related Quality of Life (Medical outcomes study short-form); MHQoL (SF-12) = Mental Health-Related Quality of Life (Medical outcomes study short-form). The final model included invariant predictive paths, which explains why the non-standardized coefficients (*b*) are invariant across time periods. Conversely, the standardized coefficients (*ß*) are expressed in standard deviation units and are thus a function of the variances of latent constructs on which no constraints were imposed, and thus differ slightly across time periods.

* *p* <. 01;

### Results Based on Lag 2 analyses

The detailed parameter estimates from the predictive models estimated across a two-year time lag are reported in the top of Table 4. In regards the relationships between HRQoL and ED pathology, as hypothesised, higher levels of ED symptoms and higher BMI independently predicted poorer PHQoL and poorer MHQoL two years later. The inverse relationships were partially supported. Poorer PHQoL significantly predicted higher levels of ED symptoms after two years but had no effect on later BMI. On the other hand MHQoL had no effect on ED symptoms or BMI after two years. In regards the relationships between PD and ED pathology, higher levels of ED symptoms and higher BMI independently predicted greater PD two years later, while greater PD predicted higher BMI but had no effect on ED symptoms.

**Table 4:** Standardized parameter estimates from the final predictive models (Two and Four Year Lag).

*Two Year Lag*		*Time 1 → Time 3*	*Time 3 → Time 5*	*Time t → Time t+2*
*Predictors (t)*	*Outcomes (t +2)*	*ß (E*.*S*.*)*	*ß (S*.*E*.*)*	*ß (S*.*E*.*)*
BMI	BMI	.843 (.025)*	.855 (.035)*	.880 (.035)*
	PD (K-10)	.117 (.030)	.166 (.041)*	.116 (.031)*
	PHQoL (SF-12)	-.121 (.035)*	-.129 (.038)*	-.199 (.062)*
	MHQoL (SF-12)	-.094 (.031)*	-.093 (.031)*	-.083 (.028)*
ED (EDEQ)	ED (EDE-Q)	.635 (.031)*	.709 (.026)*	.677 (.030)*
	PD (K-10)	.136 (.028)*	.198 (.043)*	.155 (.036)*
	PHQoL (SF-12)	-.099 (.034)*	-.109 (.038)*	-.187 (.068)*
	MHQoL (SF-12)	-.147 (.032)*	-.148 (.033)*	-.149 (.036)*
PD (K-10)	PD (K-10)	.403 (.038)*	.548 (.040)*	.402 (.034)*
	BMI	.217 (.063)*	.210 (.064)*	.227 (.070)*
	ED (EDE-Q)	-.018 (.062)	-.019 (.064)	-.017 (.057)
PHQoL (SF-12)	PHQoL (SF-12)	.392 (.050)*	.627 (.048)*	.611 (.062)*
	BMI	.028 (.028)	.042 (.042)	.028 (.028)
	ED (EDE-Q)	-.117 (.025)*	-.191 (.039)*	-.103 (.024)*
MHQoL (SF-12)	MHQoL (SF-12)	.457 (.041)*	.665 (.038)*	.704 (.052)*
	BMI	.103 (.053)	.154 (.079)	.187 (.098)
	ED (EDE-Q)	-.058 (.047)	-.093 (.075)	-.094 (.076)
*Four Year Lag*		*Time 1 → Time 5*	*Time 5 → Time 9*	*Time t → Time t+4*
*Predictors (t)*	*Outcomes (t +4)*	*ß (E*.*S*.*)*	*ß (S*.*E*.*)*	*ß (S*.*E*.*)*
BMI	BMI	.817 (.036)*	.832 (.035)*	.842 (.040)*
	PD (K-10)	.067 (.036)	.081 (.043)	.061 (.033)
	PH (SF-12)	-.170 (.046)*	-.171 (.048)*	-.234 (.067)*
	MH (SF-12)	-.049 (.041)	-.043 (.036)	-.041 (.034)
ED (EDE-Q)	ED (EDE-Q)	.575 (.032)*	.710 (.032)*	.616 (.032)*
	PD (K-10)	.158 (.038)*	.197 (.048)*	.186 (.047)*
	PH (SF-12)	-.115 (.039)*	-.120 (.042)*	-.206 (.076)*
	MH (SF-12)	-.125 (.044)*	-.115 (.041)*	-.138 (.050)*
PD (K-10)	PD (K-10)	.424 (.042)*	.488 (.043)*	.420 (.039)*
	BMI	.067 (.078)	.066 (.077)	.076 (.088)
	ED (EDE-Q)	.146 (.065)	.167 (.075)	.132 (.060)
PH (SF-12)	PH (SF-12)	.374 (.052)*	.543 (.056)*	.558 (.066)*
	BMI	-.034 (.039)	-.050 (.057)	-.038 (.043)
	ED (EDE-Q)	-.132 (.028)*	-.227 (.048)*	-.118 (.028)*
MH (SF-12)	MH (SF-12)	.496 (.045)*	.582 (.044)*	.680 (.056)*
	BMI	.035 (.072)	.047 (.099)	.058 (.121)
	ED (EDEQ)	.132 (.054)	.208 (.088)	.175 (.075)

*Note*. BMI = Body Mass Index; ED (EDE-Q) = Eating Disorder pathology (Eating Disorder Examination Questionnaire); PD (K-10) = Psychological Distress (Kessler Psychological Distress Scale); PH (SF-12) = Physical Health-Related Quality of Life (Medical outcomes study short-form); MH (SF-12) = Mental Health-Related Quality of Life (Medical outcomes study short-form). The final model included invariant predictive paths, which explains why the non-standardized coefficients (*b*) are invariant across time periods. Conversely, the standardized coefficients (*ß*) are expressed in standard deviation units and are thus a function of the variances of latent constructs on which no constraints were imposed, and thus differ slightly across time periods.

* *p* <. 01.

### Results Based on Lag 4 analyses

The detailed parameter estimates from the predictive models estimated across a four-year time lag are reported in the bottom section of Table 4. Regarding the relationships between HRQoL and ED pathology, as hypothesised, higher levels of ED symptoms predicted both poorer PHQoL and poorer MHQoL four years later. Higher BMI also predicted poorer PHQoL but had no effect on MHQoL after four years. Examining the inverse relationships, our hypotheses were partially supported. Poorer PHQoL predicted higher levels of ED symptoms after four years but had no relation to later levels of BMI, and MHQoL had no effect on either ED symptoms or BMI. In regards the relationships between PD and ED pathology, higher levels of ED symptoms predicted greater PD, whereas BMI had no effect on PD after four years. On the other hand, greater PD had no effect on either ED symptoms or BMI after four years.

## Discussion

The present study used a large longitudinal community sample of women to empirically test relationships between the constructs of ED pathology and QoL across time. To date the literature has been biased toward investigation of the impact of ED pathology on QoL, with little to no exploration of possible inverse relationships. To our knowledge, this is the first study to longitudinally test bidirectional influences between these constructs.

Our main hypothesis for a bidirectional relationship between ED pathology and QoL was globally supported. More precisely, we predicted and found evidence that the presence of ED pathology predicted reductions in HRQoL over time, as was also found previously by Wade and colleagues [16], as well as increased levels of PD. However, we also found that low levels of QoL in and of itself predicted increased levels of ED pathology over time, a relationship that until now had not been investigated. A further aim was to examine the time interval required to observe these relationships, and to assess their stability and longevity. We found evidence for a bidirectional relationship between QoL and ED pathology at time lags of one year, suggesting that the impact of QoL on ED pathology and vice versa is observable within at least 12 months. As evidence for the longevity of the QoL-ED relationship, we continued to find evidence for reciprocal predictive relationships between QoL and ED pathology over a time period of up to four years. Finally, evidencing stability of the QoL-ED relationship, we observed signs of this bidirectional relationship over four separate one-year time periods, two separate two-year periods, and two separate four-year periods.

However, although evidence for bidirectional relationships was globally evident across all periods and lags considered, some specific relationships between HRQoL and ED pathology changed across the time lags considered. Across time lags of one, two, and four years, having higher levels of ED symptoms and a higher BMI consistently predicted a worsening of HRQoL. Conversely, lower levels of physical HRQoL consistently predicted later increases in ED severity across the time lags considered. On the other hand, lower levels of mental HRQoL only predicted increases in ED severity across a one-year time interval. The particularly strong bidirectional relationship between physical HRQoL and ED pathology in this study may be partly attributable to the type of ED pathology present in the samples, which as may be expected in a community sample [55], have been found to be more characteristic of bulimic (versus anorectic) eating disorders and associated with a higher than average BMI [56]. Considering the impact of ED symptoms on physical HRQoL, behaviors such as binge eating are linked to excessive weight gain and associated physical health problems [57]. In regards to the impact of poor physical HRQoL on ED symptoms, being overweight in a society that idealises weight loss may be expected to trigger excessive preoccupation and dissatisfaction with one’s body weight/shape [58] and lead to maladaptive eating patterns such as periods of fasting punctuated by regular binge eating.

Alongside functional impairment, the DSM also posits distress as essential to defining major mental disorders [1]. Both these constructs are also highlighted as important aspects of QoL [3]. Our analyses allowed us to independently assess the impact of psychological distress and HRQoL on ED pathology and vice versa. In contrast to physical HRQoL, psychological distress only predicted increased levels of ED symptoms in the shorter term, and similarly to mental HRQoL did not continue to have an effect after 12 months. Thus the perception of poor physical health that limits the ability to function optimally within social and occupational roles appeared to contribute more to the later development or exacerbation of eating and body image disturbances than the experience of general psychological distress. Conversely, ED symptoms were found to contribute to both increased psychological distress and reductions in HRQoL consistently over time, and this highlights what has already been widely discussed regarding the long-lasting negative impact of disturbances in body image and eating behaviours on QoL [2].

One finding that was inconsistent with our hypotheses was that ED symptoms and higher BMI predicted improvements in mental HRQoL after a one year interval. These unexpected relationships did not last however, and the analyses based on two and four year time lags demonstrated that the relationships between these variables in both directions were consistent with our expectations. Given that the observed correlations between these constructs (see S1 Analyses) were in the expected direction, a possible explanation for this unexpected result observed in the one-year time lag analyses could be related to the nature of the models that we used, which simultaneously include multiple predictors and outcomes. Whilst a careful examination of all models considered in this study revealed no evidence that multicollinearity problems may have impacted the results, the effects of each predictor was estimated as net of what this predictor shared with the other predictors, and reflects the influence of this predictor on the level of outcomes net of what they share with the other outcomes. More precisely, this meant that the effects of ED symptoms and BMI on mental HRQoL reflected their impact on this outcome over and above their more marked impact on physical HRQoL and psychological distress. This suggests that ED symptoms may have a slight short-term positive effect on mental HRQoL. Indeed, both egosyntonic (e.g. dietary restriction) and egodystonic (e.g. binge eating) ED symptoms may be used as ways to cope with life difficulties by providing a perception of control or method of self-soothing in the short term (e.g., [14]). While the current results may be suggestive of this, they also indicate that any beneficent effect of ED symptoms is not lasting, and over the longer-term ED symptoms were found to be associated with both poorer physical and mental HRQoL and greater psychological distress. Clearly, future research is warranted to clarify this relationship and ascertain the true nature of the relationship between HRQoL and ED symptoms within a one year timeframe.

### Clinical Implications

Unlike almost all other disorders listed in the DSM, the EDs do not include a criterion requiring the symptoms to result in clinically significant distress and/or impairment in important areas of functioning [1]. The current study however adds to the growing literature demonstrating that varying levels of ED pathology result in both increased distress and suboptimal functioning in important areas of life [2]. Thus future revisions of the DSM may consider the addition of a ‘distress and impairment’ criterion to ED diagnoses.

The findings that lower QoL contributes to a worsening of ED symptoms over time have implications for how EDs are classified and for clinical practice. Owing to findings that ED symptoms result in a worsening of QoL, calls have been made for QoL to be measured as an important outcome of ED treatment [2], since successful treatment of an ED should also resolve the impact of symptoms on other domains of life. However, the findings in the current study also suggest that in and of itself, poorer QoL contributes to ED symptomatology. Thus there are two reasons why clinicians may consider implementing interventions that specifically target QoL in the treatment of patients with EDs: (i) because improving QoL should at least partially contribute to the resolution of ED symptoms; and (ii) because remission from an ED may not fully resolve poor QoL (e.g., [59,60]).

Embracing QoL as a treatment target rather than just an outcome entails a re-conceptualisation of the relationship between QoL and EDs, which currently is that poor QoL is expected to be rectified by addressing ED symptoms. Whilst the dominant models of ED treatment remain highly centred on targeting ED symptoms (e.g., [61]); moving away from this symptom-centric approach is not completely novel, and was pursued by [62] in her pioneering psychotherapeutic treatment of both obesity and anorexia nervosa in the 1960s and 70s. Debate continues around this issue with some clinical experts calling for a reduction in the weight-centric focus of ED clinicians [63] and others for a broader definition of recovery [64]. Further to this, considering that psychological comorbidities are common in EDs (e.g., [65]), and that psychological distress in the current study independently predicted an increase in ED severity after one year, a broader approach to treating patients with EDs may be enhanced by also reducing the impact of non-ED symptoms, such as those related to anxiety or depression.

Recent trials are suggestive of such a broader approach. In a randomised controlled trial of three therapies to treat anorexia nervosa, McIntosh and colleagues [66] unexpectedly found that SSCM (Specialist Supportive Clinical Management), developed as an active control therapy for the comparison of CBT and interpersonal psychotherapy (IPT), outperformed both CBT and IPT in improving both ED symptoms and global functioning. In a study by Williams and colleagues [14], a Community Outreach Partnership Program (COPP) was developed in order to enhance the QoL of people with EDs who did not respond to more traditional symptom-focused approaches. Evaluation of COPP found that, despite ED recovery not being an explicit aim of the program, significant improvement was observed in ED symptoms following four months of therapy. Finally, in a recent randomised controlled trial comparing CBT-SE and SSCM-SE, both modified to make QoL improvements the main aim in the treatment of participants with chronic anorexia nervosa, significant improvements were found in both HRQoL and ED symptoms [17]. On the other hand, Fairburn and colleagues [67] found that only a subgroup of participants, those with more complex EDs, responded better to a broader version of enhanced CBT for EDs (CBT-Eb) compared to the more widely used focused version (CBT-Ef). The defining feature linking SSCM, COPP, and CBT-Eb is that while all include to some extent a focus on weight restoration and normalisation of eating, they also explicitly address other non-ED problems and QoL, either as directed by the client (as in SSCM and COPP) or through set treatment modules based on characteristics thought to maintain ED pathology (e.g., low self-esteem and perfectionism, as in CBT-Eb).

### Strengths and Limitations

The strengths of this study were the longitudinal design, use of a community-based sample, the standardised assessment of HRQoL, psychological distress, and ED symptoms at multiple time points over a nine year period, and the use of latent variable models to test relationships. This allowed for a sophisticated first investigation into the bidirectional relationships between EDs and QoL. Using a community-based rather than clinical sample provided more confidence in the generalizability of the findings, as it is well known that the vast majority of people with EDs do not seek treatment [19,68,69], and thus clinical samples are unlikely to be representative of the broader ED population. On the other hand, the implications for clinical practice described above need to be circumscribed by the use of a community rather than clinical sample. Similarly, this study is also limited by the fact that the sample did not include men and that the majority of participants were from a university setting. However, unlike many university samples that are often confined to undergraduate psychology students from large metropolitan institutions, the participants recruited in this study included students of various disciplines, as well as academic and professional (e.g., administration) staff who attended regional and less academically selective institutions. James Cook University for instance, which provided the majority of participants in this study, is an institution located in regional Queensland with around one in five students being of lower socioeconomic origin. Further, 122 of the participants in this study were selected from the electoral roll. Overall this recruitment strategy created a broader representation of the Australian population in terms of age, ethnicity, socioeconomic status, and education levels than is usually observed in university samples of single disciplines. However, it should be kept in mind that this study relied on a convenience sample of unknown generalizability, which also precluded the use of weights in the analyses, which would have allowed us to obtain parameter estimates unbiased from the oversampling of participants with eating disorder pathology. As such, the present results must remain preliminary, and should be replicated on more representative and properly weighted samples.

Another limitation of the current study was the use of the self-report version of the EDE (i.e., the EDE-Q). Whilst the interview version is generally considered a stronger measure of ED pathology; the questionnaire was selected since the sample was followed up by means of a postal survey. Furthermore, the EDE-Q has high convergent validity with the EDE and has been shown to be reliable and valid in detecting EDs in diverse community samples (e.g., [23,70]).

### Conclusions

The current study found evidence for a bidirectional relationship between QoL and ED pathology. This expands current knowledge on QoL in EDs substantially, as previous research has been biased toward the impact of EDs on constructs such as HRQoL and psychological distress. It is recommended that clinical research should pursue the investigation of broader treatments that target not only ED symptoms but also disturbances in other aspects of life outside of the ED.

## Supporting Information

S1 Analyses The online supplement provides additional technical information regarding confirmatory factor analyses, alternative measurement models, latent variable correlations, and proportions of variance explained (R^2^) in the outcome variables. (DOCX)
