# Supplementary material for: The Bidirectional Relationship between Quality of Life and Eating Disorder Symptoms: A 9-Year Community-Based Study of Australian Women
Source: PLoS One. 2015 Mar 26;10(3):e0120591. doi: 10.1371/journal.pone.0120591 (PMC4374670; doi:10.1371/journal.pone.0120591)
Supplement: S1 Analyses — (DOCX) [file pone.0120591.s001.docx]

**Online Supplemental Materials for:**

**The Bidirectional Relationship between Quality of Life and Eating Disorder Symptoms: A 9-Year Community-Based Study of Australian Women**

**Authors’ note:**

These online technical appendices are to be posted on the journal website and hot-linked to the manuscript. If the journal does not offer this possibility, these materials can alternatively be posted on one of our personal websites (we will adjust the in-text reference upon acceptance).

We would also be happy to have some of these materials brought back into the main manuscript if you deem it useful. We developed these materials mostly to provide additional technical information and to keep the main manuscript from becoming needlessly long.

***Confirmatory Factor Analyses***

We first verified the adequacy of the a priori longitudinal measurement model across all time waves considered here. Thus, we estimated a confirmatory factor analytic model including, at each time point, four a priori factors reflecting ED, PD, MHQoL, and PhQoL. The SF-12 measurement model was operationalised according to the solution retained by Fleishman, Selim, and Kazis (2010) in which different scoring algorithms where compared. In their final solution, the MCS and PCS factors are both defined on the basis of 7 items each, with two items specified as contributing to both factors (item 1 “General health: *In general would you say your health is*…” and item 10 “Vitality: *How much of the time during the past four weeks did you have a lot of energy*…”) with the remaining 10 items being uniquely associated with a single factor. The other factors based on the K-10 and EDE-Q were specified as congeneric, with each item allowed to load on a single factor. This longitudinal measurement model thus included a total of 24 correlated factors (4 factors * 6 time points [Waves 1, 2, 3, 4, 5, and 9] = 24). All factors were allowed to correlate within time-points as well as across time-points. In any longitudinal model, a priori correlated uniquenesses between matching indicators utilized at the different time-points, reflecting the fact that indicators’ unique variance emerges in part from shared sources of influences over time, need to be included to avoid converging on biased and inflated stability estimates (Jöreskog, 1979; Marsh, 2007).

Critical assumptions of longitudinal models are that the measurement models underlying the constructs are invariant over time and that the predictive system has reached equilibrium (e.g., Cole & Maxwell, 2003). The first assumption tests whether the constructs are defined in the same manner across time points (Meredith, 1993; Millsap, 2011), whereas the second assumption tests whether the overall pattern of associations between the constructs remains stable across time periods – supporting the idea that the results are not a function of time-specific events but can be expected to generalize to different time periods (e.g., Cole & Maxwell, 2003). Tests of measurement invariance across time points were conducted first in the following sequence (Meredith, 1993; Millsap, 2011; Morin et al., 2011): (i) configural invariance (the same measurement model is estimated at all time-points, with no equality constraints), (ii) weak invariance (invariance of the factor loadings); (iii) strong invariance (invariance of the loadings and thresholds; thresholds replace the intercepts in WLSMV estimation and represent the point at which answers change from one category to another); (iv) strict invariance (invariance of the loadings, thresholds and uniquenesses). The second assumption of equilibrium was tested directly within the predictive models and reported in the main manuscript.

The fit results from these confirmatory factor analyses are reported in the top section of Table S1 (also in the online supplements). These results confirm the adequacy of the a priori longitudinal measurement model estimated across all time waves with indices indicating acceptable to excellent fit (RMSEA ≤ .06; CFI ≥ .95; TLI ≥ .90). Tests of measurement invariance (see Table S1), confirmed the strict measurement invariance of this model across time (configural, loadings, thresholds, uniquenesses) as none of the changes in fit indices exceeded the recommended cut-offs of .01 for the CFI and .015 for the RMSEA. Likewise, the TLI showed no changes exceeding .008.

Parameters estimated from this strictly invariant measurement model were used to compute scale score-reliability estimates from the various factors included in this model using McDonald’s (1970) omega coefficient:

$$\omega=\frac{\left( \sum\left| \lambda_{i} \right| \right)^{2}}{\left[ \left( \sum\left| \lambda_{i} \right| \right)^{2}+ \sum\delta_{i} \right]}$$

where $\left| \lambda_{i} \right|$ are the standardized factor loadings associated with a factor in absolute values, and *δi*, the item uniquenesses. The numerator, were the factor loadings are summed, and then squared, reflects the proportion of the variance in in indicators that reflect true score variance, whereas the denominator reflects total amount of variance in the items including both true score variance and random measurement errors (reflects by the sum of the items uniquenesses associated with a factor). Compared with traditional estimates of reliability such as Cronbach’s α (see Sijtsma, 2009), *ω* provides more precise estimates of scale scores reliability by taking into account the strength of association between items and constructs (*λi*) as well as item-specific measurement errors (*δii*), whereas α simply attributes an equal weight to all indicators. Omega is interpreted like alpha coefficients (or other indicators or scale score reliability) as reflecting the proportion of true score variance in the measure (1- *ω* reflects the proportion of random measurement error) and values higher than .70 generally taken to be satisfactory for research purposes (see Sijtsma, 2009). Supporting the strength of the measurement model, these coefficients were all relatively high and satisfactory across time points: ED (*ω* = 0.968 to 0.995; *M* = 0.975; *SD* = 0.010); PD (*ω* = 0.934 to 0.964; *M* = 0.946; *SD* = 0.010); MHQoL (*ω* = 0.859 to 0.913; *M* = 0.897; *SD* = 0.020); PHQoL (*ω* = 0.835 to 0.927; *M* = 0.904; *SD* = 0.034).

**Table S1**.

*Results from the Alternative Measurement Models tested in this Study.*

| *Models* | *χ²* | *df* | *RMSEA (90% CI)* | *CFI* | *TLI* | *MDΔχ²* | *Δdf* | *ΔRMSEA* | *ΔCFI* | *ΔTLI* |
| --- | --- | --- | --- | --- | --- | --- | --- | --- | --- | --- |
| *Measurement models Times 1 to 9* |  |  |  |  |  |  |  |  |  |  |
| Configural Invariance | 39888.826* | 31928 | .017 (.017-.018) | .950 | .948 | -- | -- | -- | -- | -- |
| Weak invariance (loadings) | 40658.821* | 32132 | .018 (.017-.018) | .946 | .945 | 2821.215* | 204 | +.001 | -.004 | -.003 |
| Strong invariance (loadings, thresholds) | 42234.481* | 32897 | .019 (.018-.019) | .941 | .941 | 3745.829* | 765 | +.001 | -.005 | -.004 |
| Strict invariance (loadings, thresholds, uniq.) | 43786.632* | 33112 | .020 (.019-.020) | .933 | .933 | 1192.379* | 215 | +.001 | -.008 | -.008 |

***Note****. χ²* = WLSMV chi square; *df*= degrees of freedom; *RMSEA* = Root mean square error of approximation; *90% CI* = 90% Confidence Interval for the RMSEA; *CFI* = Comparative fit index; TLI = Tucker-Lewis index; Δ since previous model; MDΔχ^2^ : chi square difference test based on the Mplus DIFFTEST function for WLSMV estimation. With WLSMV estimation, the χ^2^ values are not exact, but "*estimated*" as the closest integer necessary to obtain a correct p-value. This explains why sometimes the χ^2^ and resulting CFI values can be non-monotonic with model complexity. Given that the MDΔχ^2^ tends to be oversensitive to sample size and to minor model misspecifications, as the chi-square itself, and to take into account the overall number of MDΔχ^2^ tests used in this study, the significance level for these tests was set at .01 (Bollen, 1989; Morin et al., 2009; Rensvold, & Cheung, 1998). * *p* < 0.01.

**Table S2**.

*Correlations Among Variables Measured in this Study.*

|  | ED1 | ED2 | ED3 | ED4 | ED5 | ED9 | PHQoL1 | PHQoL2 | PHQoL3 | PHQoL4 | PHQoL5 | PHQoL9 | MHQoL1 |
| --- | --- | --- | --- | --- | --- | --- | --- | --- | --- | --- | --- | --- | --- |
| ED1 |  |  |  |  |  |  |  |  |  |  |  |  |  |
| ED2 | 0.68^**^ |  |  |  |  |  |  |  |  |  |  |  |  |
| ED3 | 0.65^**^ | 0.68^**^ |  |  |  |  |  |  |  |  |  |  |  |
| ED4 | 0.60^**^ | 0.69^**^ | 0.79^**^ |  |  |  |  |  |  |  |  |  |  |
| ED5 | 0.62^**^ | 0.69^**^ | 0.78^**^ | 0.78^**^ |  |  |  |  |  |  |  |  |  |
| ED9 | 0.55^**^ | 0.61^**^ | 0.64^**^ | 0.66^**^ | 0.77^**^ |  |  |  |  |  |  |  |  |
| PHQoL1 | -0.37^**^ | -0.40^**^ | -.318^**^ | -0.28^**^ | -0.32^**^ | -0.33^**^ |  |  |  |  |  |  |  |
| PHQoL2 | -0.31^**^ | -0.24^**^ | -.326^**^ | -0.33^**^ | -0.30^**^ | -0.30^**^ | 0.48^**^ |  |  |  |  |  |  |
| PHQoL3 | -0.25^**^ | -0.23^**^ | -.279^**^ | -0.34^**^ | -0.36^**^ | -0.28^**^ | 0.38^**^ | 0.54^**^ |  |  |  |  |  |
| PHQoL4 | -0.26^**^ | -0.22^**^ | -.285^**^ | -0.23^**^ | -0.34^**^ | -0.31^**^ | 0.45^**^ | 0.49^**^ | 0.53^**^ |  |  |  |  |
| PHQoL5 | -0.28^**^ | -0.27^**^ | -.335^**^ | -0.31^**^ | -0.35^**^ | -0.29^**^ | 0.44^**^ | 0.51^**^ | 0.57^**^ | 0.62^**^ |  |  |  |
| PHQoL9 | -0.31^**^ | -0.25^**^ | -.329^**^ | -0.31^**^ | -0.34^**^ | -0.33^**^ | 0.44^**^ | 0.49^**^ | 0.55^**^ | 0.56^**^ | 0.69^**^ |  |  |
| MHQoL1 | -0.42^**^ | -0.28^**^ | -.288^**^ | -0.30^**^ | -0.29^**^ | -0.23^**^ | 0.51^**^ | 0.29^**^ | 0.32^**^ | 0.32^**^ | 0.33^**^ | 0.33^**^ |  |
| MHQoL2 | -0.36^**^ | -0.40^**^ | -.326^**^ | -0.43^**^ | -0.34^**^ | -0.38^**^ | 0.35^**^ | 0.45^**^ | 0.34^**^ | 0.31^**^ | 0.37^**^ | 0.35^**^ | 0.49^**^ |
| MHQoL3 | -0.30^**^ | -0.29^**^ | -.389^**^ | -0.36^**^ | -0.37^**^ | -0.30^**^ | 0.31^**^ | 0.31^**^ | 0.50^**^ | 0.38^**^ | 0.37^**^ | 0.36^**^ | 0.43^**^ |
| MHQoL4 | -0.30^**^ | -0.28^**^ | -.303^**^ | -0.39^**^ | -0.41^**^ | -0.31^**^ | 0.32^**^ | 0.32^**^ | 0.38^**^ | 0.50^**^ | 0.42^**^ | 0.43^**^ | 0.44^**^ |
| MHQoL5 | -0.42^**^ | -0.39^**^ | -.405^**^ | -0.39^**^ | -0.47^**^ | -0.39^**^ | 0.38^**^ | 0.35^**^ | 0.42^**^ | 0.46^**^ | 0.60^**^ | 0.44^**^ | 0.52^**^ |
| MHQoL9 | -0.31^**^ | -0.33^**^ | -.350^**^ | -0.36^**^ | -0.40^**^ | -0.48^**^ | 0.36^**^ | 0.37^**^ | 0.38^**^ | 0.34^**^ | 0.42^**^ | 0.56^**^ | 0.40^**^ |
| PD1 | 0.63^**^ | 0.64^**^ | 0.45^**^ | 0.46^**^ | 0.49^**^ | 0.40^**^ | -0.48^**^ | -0.15^**^ | -0.23^**^ | -0.23^**^ | -0.20^**^ | -0.20^**^ | -0.57^**^ |
| PD2 | 0.45^**^ | 0.53^**^ | 0.43^**^ | 0.44^**^ | 0.39^**^ | 0.44^**^ | -0.32^**^ | -0.38^**^ | -0.30^**^ | -0.28^**^ | -0.32^**^ | -0.30^**^ | -0.48^**^ |
| PD3 | 0.35^**^ | 0.36^**^ | 0.45^**^ | 0.39^**^ | 0.41^**^ | 0.36^**^ | -0.30^**^ | -0.26^**^ | -0.39^**^ | -0.35^**^ | -0.29^**^ | -0.34^**^ | -0.38^**^ |
| PD4 | 0.37^**^ | 0.40^**^ | 0.46^**^ | 0.49^**^ | 0.52^**^ | 0.44^**^ | -0.30^**^ | -0.36^**^ | -0.33^**^ | -0.43^**^ | -0.35^**^ | -0.37^**^ | -0.41^**^ |
| PD5 | 0.43^**^ | 0.44^**^ | 0.45^**^ | 0.45^**^ | 0.54^**^ | 0.43^**^ | -0.28^**^ | -0.28^**^ | -0.30^**^ | -0.39^**^ | -0.43^**^ | -0.35^**^ | -0.41^**^ |
| PD9 | 0.31^**^ | 0.39^**^ | 0.40^**^ | 0.40^**^ | 0.48^**^ | 0.56^**^ | -0.26^**^ | -0.28^**^ | -0.26^**^ | -0.25^**^ | -0.33^**^ | -0.41^**^ | -0.33^**^ |
| BMI1 | 0.34^**^ | 0.26^**^ | 0.24^**^ | 0.25^**^ | 0.28^**^ | 0.28^**^ | -0.32^**^ | -0.38^**^ | -0.24^**^ | -0.32^**^ | -0.27^**^ | -0.25^**^ | -0.15^**^ |
| BMI2 | 0.31^**^ | 0.29^**^ | 0.26^**^ | 0.27^**^ | 0.34^**^ | 0.28^**^ | -0.31^**^ | -0.35^**^ | -0.25^**^ | -0.35^**^ | -0.33^**^ | -0.27^**^ | -0.11 |
| BMI3 | 0.28^**^ | 0.25^**^ | 0.28^**^ | 0.33^**^ | 0.35^**^ | 0.28^**^ | -0.27^**^ | -0.41^**^ | -0.35^**^ | -0.37^**^ | -0.34^**^ | -0.30^**^ | -0.16^**^ |
| BMI4 | 0.32^**^ | 0.30^**^ | 0.32^**^ | 0.34^**^ | 0.37^**^ | 0.33^**^ | -0.30^**^ | -0.42^**^ | -0.35^**^ | -0.40^**^ | -0.35^**^ | -0.33^**^ | -0.19^**^ |
| BMI5 | 0.30^**^ | 0.28^**^ | 0.33^**^ | 0.34^**^ | 0.38^**^ | 0.35^**^ | -0.25^**^ | -0.37^**^ | -0.33^**^ | -0.40^**^ | -0.44^**^ | -0.38^**^ | -0.14^**^ |
| BMI9 | 0.33^**^ | 0.30^**^ | 0.29^**^ | 0.32^**^ | 0.38^**^ | 0.39^**^ | -0.32^**^ | -0.38^**^ | -0.31^**^ | -0.39^**^ | -0.37^**^ | -0.40^**^ | -0.13^*^ |

NB: ED = eating disorder pathology, PHQoL = physical health-related quality of life, MHQoL = mental health-related quality of life, PD = psychological distress, BMI = body mass index. The number in column and row headings denotes the time wave in years. Higher scores equate to more eating disorder pathology, greater quality of life, more psychological distress, and higher body mass index. * *p* < 0.05. ** *p* < 0.01.

**Table S2 (Continued)**.

|  | MHQoL2 | MHQoL3 | MHQoL4 | MHQoL5 | MHQoL9 | PD1 | PD2 | PD3 | PD4 | PD5 | PD9 | BMI1 |
| --- | --- | --- | --- | --- | --- | --- | --- | --- | --- | --- | --- | --- |
| MHQoL3 | 0.52^**^ |  |  |  |  |  |  |  |  |  |  |  |
| MHQoL4 | 0.52^**^ | 0.62^**^ |  |  |  |  |  |  |  |  |  |  |
| MHQoL5 | 0.53^**^ | 0.60^**^ | 0.59^**^ |  |  |  |  |  |  |  |  |  |
| MHQoL9 | 0.49^**^ | 0.46^**^ | 0.51^**^ | 0.61^**^ |  |  |  |  |  |  |  |  |
| PD1 | -0.35^**^ | -0.30^**^ | -0.37^**^ | -0.35^**^ | -0.29^**^ |  |  |  |  |  |  |  |
| PD2 | -0.77^**^ | -0.53^**^ | -0.51^**^ | -0.50^**^ | -0.52^**^ | 0.44^**^ |  |  |  |  |  |  |
| PD3 | -0.49^**^ | -0.78^**^ | -0.56^**^ | -0.52^**^ | -0.45^**^ | 0.34^**^ | 0.65^**^ |  |  |  |  |  |
| PD4 | -0.53^**^ | -0.58^**^ | -0.78^**^ | -0.54^**^ | -0.53^**^ | 0.40^**^ | 0.65^**^ | 0.68^**^ |  |  |  |  |
| PD5 | -0.47^**^ | -0.53^**^ | -0.55^**^ | -0.79^**^ | -0.56^**^ | 0.36^**^ | 0.57^**^ | 0.61^**^ | 0.66^**^ |  |  |  |
| PD9 | -0.49^**^ | -0.37^**^ | -0.44^**^ | -0.54^**^ | -0.78^**^ | 0.30^**^ | 0.56^**^ | 0.45^**^ | 0.59^**^ | 0.60^**^ |  |  |
| BMI1 | -0.10^*^ | -0.07 | -0.13^**^ | -0.21^**^ | -0.14^*^ | 0.16^**^ | 0.10^*^ | 0.02 | 0.09 | 0.16^**^ | 0.14^*^ |  |
| BMI2 | -0.05 | -0.01 | -0.11 | -.22^**^ | -0.15^*^ | 0.16^**^ | 0.06 | -0.05 | 0.09 | 0.19^**^ | 0.15^*^ | 0.90^**^ |
| BMI3 | -0.08 | -0.12^*^ | -0.13^*^ | -0.23^**^ | -0.16^**^ | 0.14^**^ | 0.07 | 0.09 | 0.10^*^ | 0.17^**^ | 0.11 | 0.78^**^ |
| BMI4 | -0.12^*^ | -0.16^**^ | -0.16^**^ | -0.25^**^ | -0.20^**^ | 0.22^**^ | 0.13^*^ | 0.12^*^ | 0.13^**^ | 0.18^**^ | 0.15^**^ | 0.81^**^ |
| BMI5 | -0.10 | -0.15^**^ | -0.19^**^ | -0.30^**^ | -0.21^**^ | 0.16^**^ | 0.10 | 0.10 | 0.15^**^ | 0.21^**^ | 0.19^**^ | 0.74^**^ |
| BMI9 | -0.08 | -0.12^*^ | -0.14^*^ | -0.28^**^ | -0.20^**^ | 0.17^**^ | 0.12 | 0.11 | 0.12^*^ | 0.20^**^ | 0.16^**^ | 0.74^**^ |

NB: ED = eating disorder pathology, PHQoL = physical health-related quality of life, MHQoL = mental health-related quality of life, PD = psychological distress, BMI = body mass index. The number in column and row headings denotes the time wave in years. Higher scores equate to more eating disorder pathology, greater quality of life, more psychological distress, and higher body mass index. * *p* < 0.05. ** *p* < 0.01.

**Table S2 (Continued)**.

|  | BMI2 | BMI3 | BMI4 | BMI5 |
| --- | --- | --- | --- | --- |
| BMI3 | 0.95^**^ |  |  |  |
| BMI4 | 0.94^**^ | 0.94^**^ |  |  |
| BMI5 | 0.92^**^ | 0.84^**^ | 0.94^**^ |  |
| BMI9 | 0.83^**^ | 0.83^**^ | 0.87^**^ | 0.90^**^ |

NB: ED = eating disorder pathology, PHQoL = physical health-related quality of life, MHQoL = mental health-related quality of life, PD = psychological distress, BMI = body mass index. The number in column and row headings denotes the time wave in years. Higher scores equate to more eating disorder pathology, greater quality of life, more psychological distress, and higher body mass index. * *p* < 0.05. ** *p* < 0.01.

**Table S3**.

*Percentages of Explained Variance (R^2^) in the Outcomes for the Predictive Models Estimated in this Study.*

| *Outcomes* | *Time 2* | *Time 3* | *Time 4* | *Time 5* | *Year 9* |
| --- | --- | --- | --- | --- | --- |
| *Lag 1 analyses* |  |  |  |  |  |
| ED (EDE-Q) | 0.327 | 0.587 | 0.789 | 0.678 | NA |
| BMI | 0.723 | 0.898 | 0.837 | 0.840 | NA |
| PHQoL (SF-12) | 0.492 | 0.619 | 0.660 | 0.623 | NA |
| MHQoL (SF-12) | 0.383 | 0.447 | 0.524 | 0.339 | NA |
| PD (K-10) | 0.553 | 0.572 | 0.478 | 0.441 | NA |
| *Lag 2 analyses* |  |  |  |  |  |
| ED (EDE-Q) | NA | 0.499 | NA | 0.662 | NA |
| BMI | NA | 0.701 | NA | 0.692 | NA |
| PHQoL (SF-12) | NA | 0.254 | NA | 0.535 | NA |
| MHQoL (SF-12) | NA | 0.328 | NA | 0.576 | NA |
| PD (K-10) | NA | 0.285 | NA | 0.491 | NA |
| *Lag 4 analyses* |  |  |  |  |  |
| ED (EDE-Q) | NA | NA | NA | 0.462 | 0.618 |
| BMI | NA | NA | NA | 0.698 | 0.734 |
| PHQoL (SF-12) | NA | NA | NA | 0.280 | 0.476 |
| MHQoL (SF-12) | NA | NA | NA | 0.337 | 0.443 |
| PD (K-10) | NA | NA | NA | 0.314 | 0.426 |

*Note.* BMI =Body Mass Index; ED (EDEQ) = Eating Disorder pathology (Eating Disorder Examination Questionnaire); PD (K-10) = Psychological Distress (Kessler Psychological Distress Scale); PHQoL (SF-12) = Physical Health-Related Quality of Life (Medical outcomes study short-form); MHQoL (SF-12) = Mental Health-Related Quality of Life (Medical outcomes study short-form).

***Additional Details on the One-Year Lag Models***

Potentially due to its important complexity, the predictive model based on a time lag of 1 year failed to converge on a fully proper solution, including out-of bound parameter estimates, negative variance estimates, etc. This suggests that this model might have been over-parameterized (e.g., [[1](#_ENREF_1)]) or simply too complex to be properly estimated with modern computing resources. Indeed, although the complete CFA based on a total of six time waves could be properly estimated, its estimation required over 300 hours on a second generation I7 processor (64 bits, quad core, overclocked to 4.2Gz, and 32Gb of RAM). Although the predictive model appear simpler, its greater parsimony (replacing many freely estimated factor correlations by a reduced number of predictive paths) made it more complex and may explain the non-convergence of the model on a fully proper solution. Thus, predictive results for the 1 year time lag were estimated using factor scores from the strictly invariant measurement model. Factors scores have the advantage of taking into account the strength of association between items and constructs. Compared to latent models, factor scores are not estimated net of measurement errors. However, this is unlikely to affect the results reported here given the high level of scale score reliability of the various constructs assessed in this study (as reported previously) and the fact that the measurement models showed strict measurement invariance (indicating that the measurement errors were stable across time). In fact, a comparison of the factor scores results with those from the fully latent model (which did not converge on a fully proper solution) yielded identical conclusions.

1. Chen F, Bollen KA, Paxton P, Curran PJ, Kirby JB (2001) Improper solutions in structural equation models: Causes, consequences, and strategies. Sociological Methods & Research 29: 468-508.
